# Molecular Detection and Differentiation of *Theileria lestoquardi*, *T. ovis* and *T. annulata* in Blood of Goats and Ticks in Kermanshah Province, Iran

**Published:** 2019-09-30

**Authors:** Mozhgan Rahmani-Varmale, Mousa Tavassoli, Bijan Esmaeilnejad

**Affiliations:** Department of Pathobiology, Faculty of Veterinary Medicine, Urmia University, Urmia, Iran

**Keywords:** *Theileria*, Tick, Goat, Nested PCR-RFLP, Iran

## Abstract

**Background:**

This study was carried out to identify *Theileria* spp. infections in goats and ticksin Kermanshah Province, western Iran from May–Sep 2015.

**Methods:**

For differentiation of different *Theileria* spp. both blood and tick samples were examined by nested PCR-RFLP.

**Results:**

Light microscopy of blood smears revealed *Theileria* spp. infection in 22 (5.5%), while 68 (17%) of blood samples were positive using nested PCR. Out of 68 positive samples, 85.3% (58/68) and 11.7% (8/68) were respectively positive for *Theileria ovis* and *T. lestoquardi*. Mixed infection was detected in 3% (2/68) cases. Overall, 420 ixodid ticks belong to seven different hard ticks species were collected from goats. *Rhipicephalus turanicus* 112 (26.7%), *R. sanguineus* 95 (22.6%), *R. bursa*, 91(21.7%), *Hyalomma anatolicum*, 55(13.1%), *H. excavatum* 27(6.4%), *H. marginatum*, 22(5.3%) and *Dermacentor marginatus*, 18(4.2%) were the main tick species infesting goats. The PCR products obtained from ticks were subjected to the differentiation of *Theileria* species. Respectively, 2 and 8 pools of *H. marginatum* and *R. turanicus* salivary glands were infected with *T. ovis* and *T. lestoquardi*. In addition, *T. annulata* and *T. lestoquardi* infection weredetected in three pools of *H. anatolicum*.

**Conclusion:**

This is the first report of goats and collected ticks to *Theileria* spp infection in Iran. The results suggest that *T. ovis* has a higher prevalence than *T. lestoquardi*. It is also postulated *H. marginatum*, *R. turanicus* and *H. anatolicum* might play an important role in the field as a vector of *Theileria* spp in this area.

## Introduction

Theileriosis is a hemoparasitic disease of domestic and wild ruminants caused by species of the genus *Theileria* that transmitted by species of Ixodid ticks. It is one of fatal diseases of sheep and goats in tropical and subtropical regions, where it causes significant economic losses as well as reduced production (1). The disease occurs due to at least six species of *Theileria* spp. including *T. ovis*, *T. separata*, *T. recondita*, *T. lestoquardi* (*T. hirci*), and *Theileria* sp. (China 1) and *Theileria* sp. (China 2) that recently was reported from north of China (1). Among *Theileria* parasites, *T. lestoquardi*, *Theileria* sp. (China 1) and *Theileria* sp. (China 2) are considered highly pathogenic (2). Two species, *T. lestoquardi* and *T. ovis*, cause ovine/caprine theileriosis in Iran (3). The presence of these two species was also confirmed by sequencing analysis and nested PCR-RLFP in Iran (4).

*Theileria annulata*, which is a causal agent of tropical malignant theileriosis in cattle, can also infect sheep (3). *Hyalomma* species have been implicated in the transmission of *T. lestoquardi* and *T. annulata*, namely *H. anatolicum*, *H. savignyi*, *H. aegyptium*, and *H. impeltatum* (5). *Rhipicephalus bursa*, *R. evretsi*, and *R. sanguineus* are considered as the main field vectors of *T. ovis* (6). Gold standard for diagnosis of theileriosis is usually based on microscopic examination of Giemsa-stained blood smears, clinical signs and the species of *Theileria* specified purely based on the animal host, which the sample was taken. On the other hand, since in most endemic areas, *T. lestoquardi* and *T. annulata* infections can occur naturally in both cross host and vectors (4, 7). The exact identification and differentiation between *Theileria* spp. parasites are important for understanding their epidemiology (8).

Achieving to identify *Theileria* species and vector ticks in goat is not feasible by using conventional *Theileria* diagnostic methods based on morphological features, especially during mixed infections and low parasitemias (4). Serological assays e.g. indirect immunofluorescence and ELISA have been developed for the laboratory diagnosis of theileriosis. However, the antibodies cannot always be detected in long-term as well as cross-reactivity of the antibodies against other *Theileria* species has limited the specificity of serological tests (7). The use of alternative techniques, such as nested PCR-RFLP molecular tool based on 18S rRNA has become necessary to provide complementary diagnostic information for low-costly and effectively identification of *Theileria* or *Babesia* species in the common host (4, 9–11). To date, earlier investigations about molecular epidemiology of ovine theileriosis in Iran restricted to eastern geographical areas and none so far has addressed the question of the *Theileria* infection in goats and vector ticks in Iran (4, 12–15).

Considering the importance of goat farming in Iran and paucity of data about species of *Theileria*, which can infect goats and field-collected Ixodid ticks, we aimed to accurate, identify *Theileria* species in goats and ticks by using microscopic examination and nested PCR-restriction fragment length polymorphism (RFLP) screening assay.

## Materials and Methods

### Field study area

The study was carried out during tick active season, from early May through late Sep 2015, in Kermanshah Province (an area of more than 24640km^2^), located in an important livestock production region and tropical area in western Iran between 33°37'–35°17' N latitudes and 45°20'–48°1' E longitudes and 1420 above sea level. Ecologically, this area is classified as a semi-arid zone. Goat rising is a very important economically occupations in this province.

### Animals, blood sampling, collection and examination of blood smears and ticks

Forty goat flocks were randomly selected by the local veterinary service of Kangavar, Songhor, Kermanshah, Gilan-e Gharb and Qasr-e Shirin areas and 400 peripheral blood samples were collected and then immediately thin blood smears prepared from ear capillaries were fixed in methanol for 5min and stained in 10% Giemsa solution in phosphate buffer solution (PBS), pH 7.2, for 20min and examined under a magnification of 1000x for the presence of intracellular forms of the parasite with morphology similar to *Theileria* spp. The percentage of infected red cell per 100 red blood cells was calculated. For estimating parasitemia, 100 microscopic fields containing approximate 1000 red blood cells per field were reviewed and the number of parasites per 100,000 red blood cells was enumerated. Even the presence of a single piroplasm was considered as positive. From each flock, at least 8 animals were randomly chosen. During the sampling, data on the variable parameters of the flock and animals were recorded. Based on size, the flocks divided into flocks with 15–50 goats and flocks with more than 50 goats. Age was the individual-level factor. In each flock, goats were categorized into two age classes (<1yr old versus ≥1yr old). Flocks were divided into two categorized: flock with tick infestation and no tick infestation. At the same location where blood samples were collected, ticks were collected by inspecting the whole body of each goat for the presence of ticks, mainly on their ears, along their nape of neck, perineum, and udder/orchid, inner thighs, shoulder region and tail base. Ixodid ticks were manually collected from the body surface of examined animals by rubbing alcohol pads surrounding the skin to remove embedded living ticks (16). The hard ticks were placed into labeled glass vials with 70% ethanol (Merck, Germany) and transferred to the parasitology laboratory. Tick species were identified using taxonomic keys (17, 18). Whole tick were washed once in Roccal (1% benzalkonium chloride) and three times in 70% ethanol and then dried. The ticks’ salivary glands were dissected out and placed into 200μl lysis buffer containing 20μl proteinase K, incubated for 10min at 55 °C. After adding of 360μl binding buffer and incubation for 10min at 70 °C, 270μl ethanol (100%) was added to solution, and after vortexing, complete volume was transferred to the MBST column. The MBST column was first centrifuged and then washed twice with 500μl washing buffer. Finally, DNA was eluted from the carrier with 50μl elution buffer and stored at –70 °C until use. They were divided into 55 pools comprising 4–10 tick specimens according to the source species and bioclimatic zones.

### DNA isolation and nested PCR

Total DNA was extracted from blood and tick samples using molecular biological system transfer kit (MBST Iran). Briefly, 200μl blood and tick samples were first lysed in 180μl lysis buffer and the proteins were degraded with 20μl proteinase K for 10min at 55 °C. After addition of 360μl bindings’ buffer and incubation for 10min at 70 °C, 270μl ethanol (100 %) was added to the solution and after vortexing, the complete volume was transferred to the MBST column. The MBST column was first centrifuged and then washed twice with 500μl washing buffer. Finally, DNA was eluted from the carrier with elution buffer. Purity of DNA was tested spectrophotometrically at wavelength of 260 and 280nm. Extracted DNA of *Theileria* was diluted to give a final concentration of 50ng.

A nested PCR was used to detect *Theileria* spp. DNAs. The homologous and variable regions of 18S rRNA gene were amplified by employing two-pair primers. Outer primers for the primary PCR were forward strand primer Thei F1 5'-AAC CTG GTT GAT CCT GCC AG-3' and reverse strand primer Thei R1 5'-AAA CCT TGT TAC GAC TTC TC-3'. The amplicon size of the primary PCR was 1700bp. The nested inner primers were forward strand primer Thei F2 5'-TGA TGT TCG TTT YTA CAT GG-3’, and reverse strand primer Thei R2 5'-CTA GGC ATT CCT CGT TCA CG-3'. After the second PCR, the size of nested PCR products for different species ranged from 1417 to 1426bp. The primer's specificity and sensitivity was assessed (11). Primary PCR was performed in a 30μl total reaction volume containing 3μl DNA(45–150 ng), outer primer (20pg), dNTP (250μM of each deoxynucleotide triphosphates), 10X PCR buffer (100mM Tris-HCl (pH 9), 500mM KCl, 1% Triton X-100), Taq polymerase (1.25U, Promega Madison, WI, USA), and MgCl2 (1.5 mM) in an automated thermocycler (Corbet Research, Australia) under following program: initial denaturation stage (5min at 94 °C), 25 cycles (denaturation step, 30 sec at 94 °C, annealing step, 30sec at 51 °C, extension step, 30sec at 72 °C) and final extension of 5min at 72 °C. Fifty ng of PCR products were used as template in nested PCR. In this stage, the amplification mixture was the same as that used in primary PCR, except that the inner primers were used. Nested PCR conditions were at 94 °C for 2min followed by 30 cycles of 94 °C for 30sec, 52 °C for 30sec 72 °C for 30sec, and final extension, 5min at 72 °C. Then, 10μl aliquots of the PCR products were stained with cyber green solution and electrophoresed through a 1.5% gel. After electrophoresis, amplified samples were visualized by UV transilluminator (BTS-20M, Japan). Expected PCR products for the different *Theileria* species are shown in Table 1.

**Table 1.** Expected PCR products for different *Theileia* spp (11)

**Species**	**The size of nested-PCR product**
***T. lestoquardi***	1417bp
***T. annulata***	1420bp
***T. ovis***	1426bp

The extracted DNA from salivary glands of ticks were amplified according to the protocol was previously described for blood samples. The positive DNA from *Theileria* spp. schizont was kindly provided by Razi Vaccine and Serum Research Institute (Tehran branch, Iran) (Accession numbers: KF429799, MG 208059, KP019206). Sterile water was served as negative control.

### Restriction enzyme analysis

The PCR product was purified using the MBST PCR product purification kit. 10–15μl of purified PCR product were then digested with 1ml (10U) of HpaII and HaeII restriction enzymes in 3μl 10× buffer and 5μl H_2_O at 37 °C for 2h according to supplier recommendations (Jena Bioscience, Jena, Germany), and analyzed using 2% agarose gel(Jena Bioscience, Jena, Germany), and analyzed using 2% agarose gel. The restriction analysis patterns for *Theileria* spp. are listed in Table 2.

**Table 2.** The pattern of RFLP of PCR products of different *Theileria* spp by using of HpaII and HaeII (13)

**Species**	**HpaII**	**HaeII**
***T. lestoquardi***	900-, 278-, 106-, 94- and 39bp	No digestion
***T. annulata***	1178-, 106-, 94- and 39bp	N digestion
***T. ovis***	856-, 326-, 204-and 39bp	295 and 1131bp

### Statistical analysis

The Fisher’s exact test and Mantel-Haenszel test were used to show association between the presence (positive and negative blood samples) of *Theileria* and the various parameters i.e. flock size, gender and age of animal, tick infestation of goats and presence of ticks in the flock. McNemar’s chi-square test was used to compare the data of blood smears with blood PCR method. Results were displayed as P-values as well as relative risk values (with 95% confidence intervals). P< 0.05 was accepted to be statistically significant.

## Results

Microscopic examination of thin blood smears showed parasitemia in infected animals ranging from 0.011% to 0.012% piroplasms. Low numbers of highly polymorphous parasites inside the red blood cells were detected in most of the blood smears. Samples with round, oval, ring and anaplasmoid forms were tentatively classified as *Theileria* spp. Microscopic examination of 400 blood smears obtained from five different areas of Kermanshah Province revealed that 22 (5.5%) goats were positive for piroplasms. The percentage of positive smears for theileriosis was determined between 1.25% and 10% in this region. The highest cases of *Theileria* piroplasmosis was seen in Qasr Shirin (10%), followed by Gilan Gharb (8.75%), Kermanshah (5%), Kangavar (2.5%) and Songhor (1.25%) (Table 3).

**Table 3.** Results of microscopic examination and nested PCR and RFLP for *Theileria* spp in different areas of the Kermanshah Province

**Area**	**ME^a^**				**Nested PCR**		***T. ovis***		***T. lestoquardi***		***T. annulata***		**Mixed**	

	**NS^b^**	**P^C^**	**%**	**Parasitemia (%)**	**P**	**%**	**p**	**%**	**p**	**%**	**p**	**%**	**p**	**%**
**Kangavar**	80	2	2.5	0.011	10	12.5	10	100	0	0	0	0	0	0
**Songar**	80	1	1.25	0.011	5	6.25	5	100	0	0	0	0	0	0
**Kermanshah**	80	4	5	0.011	14	17.5	14	100	0	0	0	0	0	0
**Gilan-e-gharb**	80	7	8.75	0.011	18	22.5	14	77.8	3	16.7	0	0	1	5.5
**Qasr-e-shirin**	80	8	10	0.012	21	26.25	15	71.4	5	23.9	0	0	1	4.7
**Total**	400	22	5.5	0.011	68	17	59	85.3	8	11.7	0	0	2	3

a Microscopic examination,

b Number of sample,

c Positive sample

**Fig. 1.**
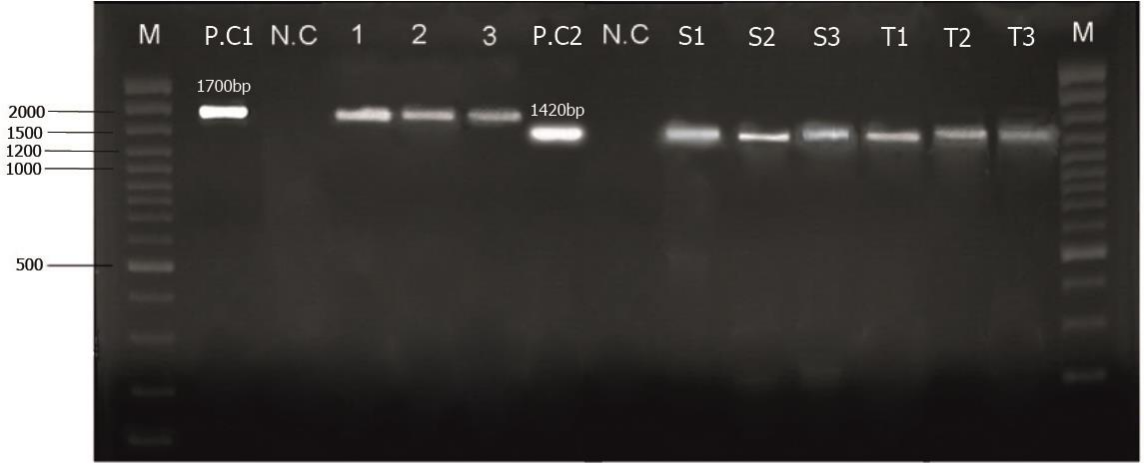
Product of *Theileria* spp using primary PCR and nested inner primers. Lane M: Molecular marker (100bp plus ladder); lane P.Ct1and PCt2: positive controls; lane N.C: negative control, lanes 1–2 and S1–S3: PCR products from infected goats; lane 3and T1–T3: PCR products from infected adult ticks*.*

The results of nested PCR assay showed that 17% (68/400) of the goats were infected with *Theileria* spp. (Table 3, Fig. 1). The highest rate of infection was observed in Qasr-e Shirin, 26.25% (21/68), that was followed by Gilan Gharb, Kermanshah, Kangavar, and Songor, 22.5% (18/68), 17.5% (14/68), 12.5% (10/ 68), and 6.25% (5/68), respectively. RFLP analysis proved presence of *T. ovis* in majority (85.3%) of *Theileria* positive samples, while *T. lestoquardi* and mixed infection was detected in 11.7% (8/68) and 3% (2/68) of the samples, respectively. Moreover, enzymatic digestion of PCR products of mixed infections revealed that 1 sample was infected with *T. lestoquardi* and *T. ovis*, and one sample with *T. lestoquardi* and *T. annulata*. All microscopically positive samples were confirmed by nested PCR. No *Theileria* piroplasms were seen on blood smears of samples that were negative in nested PCR. However, there were 46 PCR positive samples, which were negative in microscopic examination (Table 4).

**Table 4.** Comparison of microscopic examination and nested PCR analysis results in *Theileria* infection diagnosis in goats

**Result**	**Test**	

	**Thin blood smear**	**Nested PCR**
**Positive**	22	68
**Negative**	378	332
**Total**	400	400

The prevalence of *Theileria* spp. infection in age groups and different gender were not significantly different (Table 5).

**Table 5.** Association between the presence (PCR-positive and negative blood samples) of *Theileria* spp infection in goats and the evaluated parameters (flock size, age, gender and tick burden of animal)

	**Flock Size**		**Age of animal**		**Gender og animal**		**Tick burden of animal**	

	**15–50 animals**	**>50 animals**	**<1 year**	**>1 year**	**Male**	**Female**	**No tick**	**Tick infestation**
**Number**	210	190	100	300	85	315	305	95
**Negative**	17.7 (84.2%)	155 (81.5%)	84 (84%)	248 (82.4%)	70 (82.4%)	262 (83.2%)	274 (89.8%)	58 (61%)
**Positive**	33 (15.8%)	35 (18.5%)	16 (16%)	52 (17.4%)	15 (17.6%)	53 (16.8%)	31 (10.2%)	37 (39%)
P(F)*	P(F)= 0.59 (NS)		P(F)= 0.88 (NS)		P(F)= 0.40 (NS)		P(F)= 0.007	

* P (F): Fisher's exact test, P value, Ns: not significant

In this study, 420 ticks of seven different species, *R. turanicus* 112 (26.7%), *R. sanguineus* 95 (22.6%), *R*. *bursa* 91 (21.7%), *H. anatolicum* 55(13.1%), *H. excavatum* 27 (6.4 %), *H. marginatum* 22 (5.3%) and *D. marginatus*, 18(4.2%) (Table 6) were isolated and then analyzed using nested PCR in terms of the presence of *Theileria* spp. infection.

**Table 6.** Frequency of tick species collected on the body of goats

**Tick species **	**Tick number (%)**	**Tick pool of salivary glands**	

		**(No. of male)**	**(No. of female)**
***R. teranicus***	112 (26.7%)	8 (84)	3 (28)
***R. sanguineus***	95 (22.6%)	6 (60)	5 (48)
***R. bursa***	91 (21.7%)	5 (54)	4 (41)
***H. anatoliaum***	55 (13.1%)	4 (39)	2 (19)
***H. excavetum***	27 (96.4%)	2 (17)	1 (10)
***H. marginatum***	22 (5.3%)	2 (18)	1 (4)
***Dermacentormargintus***	18 (4.2%)	1 (12)	1 (6)
**Total**	420	28 (284)	17 (156)

There are 45 tick pools, of which, 13 pools that belong to the salivary glands of *H. marginatum*, *R. turanicus*and *H. anatolicum*were positive for *Theileria* spp. infection (Table 7) (Fig. 1). The frequency of *Theileria* spp. infection was higher in flocks with tick infestation than no tick infestation (P< 0.05). Frequency of *Theileria* spp. infection was significantly (P< 0.05) higher in female ticks.

**Fig. 2.**
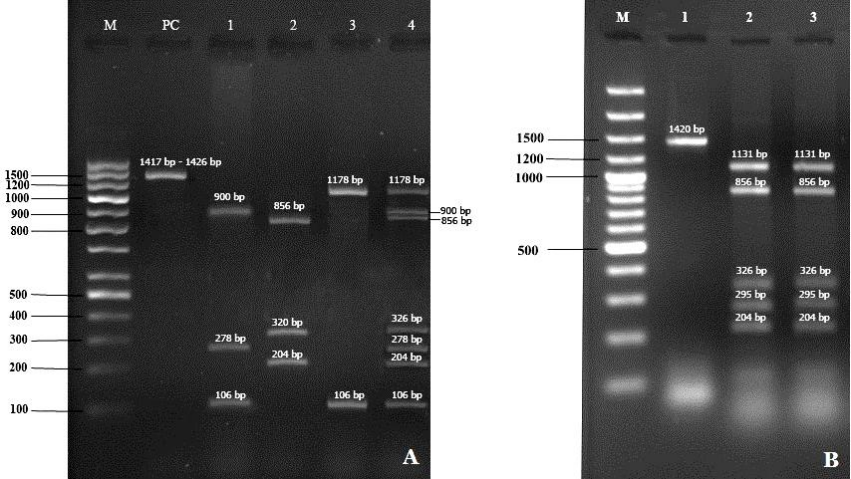
RFLP patterns of *Theileria* spp amplification products. (A) Lane M: molecular marker (100bp plus ladder), lane PC, undigested PCR product, Lane 1: *T. lestoquardi* HpaII digest, lane 2: *T. ovis* HpaII digest, lane 3: *T. annulata* HpaII digest, lane 4: mixed *T. lestoquardi*, *T. ovis* and *T. annulata* with HpaII. (B) Lane M: molecular marker (100bp plus ladder), lane 1: undigested PCR product, lane 2: *T. ovis* HaeII digest isolated from infected goats; lane 3: *T. ovis * HaeII digest from infected tick.

Molecular identification results by nested PCR-RFLP screening technique demonstrated that at least three genetically distinct *Theileria* spp. are found in both blood and ticks samples in surveyed-area. Digestion of PCR product sobtained from goats and ticks, which were positive for Theileria spp. using HpaII and HaeII, was shown in Table 2. Out of 68 positive samples, 85.3% (59) and 11.7% (8) were positive for *T. ovis* and *T. lestoquardi*, respectively. Mixed infection was detected in 3% (2) cases. Digestion of PCR products obtained from animals revealed that one sample with *T. ovis* and *T. lestoquardi*, as well as one sample with *T. lestoquardi* and *T. annulata* mixed infections were detected (Table 3, Fig. 2). Respectively, 2 and 8 pools of *H. marginatum*and *R. turanicus*salivary glands were infected with *T. ovis*and *T. lestoquardi* (Table 7).

**Table 7.** Results of molecular methods for detection *Theileria* spp in salivary glands of Ixodid ticks

**Tick species**	***T. ovis***		***T. lestoquardi***		***T. annulata***		**Total (%)**

	**No. of male (%)**	**No. of female (%)**	**No. of male (%)**	**No. of female (%)**	**No. of male (%)**	**No. of female (%)**	
***Hyalomma marginatum***	1 (1)	1 (2)	-	-	-	-	2 (3)
***Rhipicephalus turanicus***	1 (1)	3 (21)	1 (3)	3 (20)	-	-	8 (45)
***Hyalomma anatolicum***	-	-	-	1 (2)	1 (1)	1 (14)	3 (17)
**Total**	2 (2)	4 (23)	1 (3)	4 (22)	1 (1)	1 (14)	13 (65)

## Discussion

The highest infection rate of *Theileria* in Qasr Shirin and Gilan Gharb may probably be associated to that these regions bordered with Iraq where has been known as a foci of this disease. This finding is the closest to other results (19, 20) that have reported 28.8% and 20.8% of *Theileria* spp. infections in Iraq, respectively. Moreover, several studies performed to detect *Theileria* infection by the microscopic method in goats in different geographical regions of Turkey, western neighbor of Iran (21–23). 3.8% of sheep and goats blood samples were *Theileria* positive in Pakistan by microscopy observation (24). In Italy, the prevalence of *Theileria* infection was 1.6% (25). The results concerning the lower incidence of *Theileria* infection in the goat are compatible with the results in this paper. The reason that the goat had a low prevalence of *Theileria* infection may probably be ascribed to the ability of the goat to graze in unreachable and inclined areas. In this situation, goat is less exposed to the bite of infected-ticks (25). However, in contrast with the results collected in this paper regarding the low prevalence of *Theileria* infection in goat, the high-frequency rates of *Theileria* spp. infection in goats were reported from various northern zones of Egypt (85.33%) and Iran (66.7%) (26).This difference is likely due to the differences between surveyed-areas and sampling design as the present work sampled-goats were selected randomly.

In the present study, the parasitemia was not exceeded by 10.012%. In similar studies, (22, 27, 28) goat infected with *Theileria* spp*.* commonly had low parasitemia. It could be attributed to first, in caprine benign theileriosis, this normally gives very low parasitaemia and second, *T. lestoquardi* is considered as a pathogenic parasite that causes the death of animals before the appearance of the parasite in lymphoid cells and erythrocytes (29).

In Iraq, serological tests employing Indirect Fluorescent Antibody Test (IFAT) were used and the seropositivity rate of *T. lestoquardi*in goats varied from 7.3% to 3.8% in different regions of the country (30, 31). Moreover, using IFAT, 8.9% infection of goats by *Theileria* were reported in Turkey (22). In the present study, covering five different regions of Kermanshah Province, the molecular prevalence ranged from 6.25% to 26.25%. Although the results of the present and previous study cannot be compared due to the different methods were employed, the results clearly indicated that theileriosis was not broadly dispersed in this region.

In the present study, the age and sex of the animals did not show any significant association with *Theileria* spp. infection. In the endemic area, due to the relatively high number of infected ticks found and the fact that young and old goats are being continuously exposed to infected ticks, a stage of enzootic stability may have been developed (32). These results are in agreement with the finding of other researchers (33, 34). On the contrary, age and sex influenced the prevalence of piroplasmosis. These two contradictory findings may be attributed to the difference in the number of examined animals, natural immunity, pregnancy, drug administration for controlling of hemoprotozoan and recent use of acaricides, which had an effect on tick distribution (15, 35).

As expected, the prevalence of *Theileri*a spp. infection in goats detected by nested PCR was significantly (P< 0.05) higher than that obtained in microscopic examination of thin blood smears. Therefore, DNA amplification methods had higher efficiency than microscopic examination for detection of *Theileria*. The results were in agreement with a previous report about ovine theileriosis (11, 36). This study also revealed that recovered animals frequently sustain subclinical infections, which are microscopically undetectable.

Due to no report on caprine theileriosis in Iran to compare with the present study, therefore the data was comparable to the previous studies on the prevalence of *Theileria* spp. infection in goats in other countries. In previous similar studies done in other countries in Middle East e.g. Turkey (10, 21, 37) and Syria (38), *T. ovis* is reported as the most prevalent species. The same result was obtained from Tunisia as well (39). In disagreement with these findings, in Iraq, the most abundant *Theileria* species identified was *T. lestoquardi* (19, 30)*.* The dissimilarity amongst results of the two studies may be related to the different diagnostic methods that they used for microscopic examination to assess the frequency of *Theileria* spp. infection in goats; obviously molecular methods are most reliable.

The low prevalence of *T. lestoquardi* found in this study is in concern with those reported earlier (10, 21). The lower prevalence of *T. lestoquardi* infection found in goats might be because of *H. anatolicum*, which is the natural vector of this *Theileria* species (23). Although this species is widely distributed all over Iran, low infestation 13.1% (55/420) was recorded during this survey, probably because goats may not be a preferable host for *H. anatolicum anatolicum* (40). Most of the malignant small ruminant theileriosis foci in Iran are situated in places with a mean annual temperature (MAT) between 20–25 °C that often are under latitude (L) of 30° in the south part of Iran (41). In this situation, parasite can infect the goats but failed to reach infective numbers, subsequently, less infection occurs (27). The high prevalence of *T. lestoquardi* was reported from Qasr Shirin as 23.9%. This result may probably be attributed to that Qasr Shirin county situate in nearly of most famous endemic foci of malignant small ruminant theileriosis in the south of Iran such as Ahvaz and Ilam with same geographical and climatic features (4).

*Theileria annulata* is transmitted by *H*. *anatolicum* in Kermanshah Province, Iran (42), and in most of these areas both cattle and goat are raised close to each other, our findings regarding infections of *T. annulata* in goat are reasonable. The presence of antibodies against *T. annulata*was demonstrated earlier in infected goats (43). In the present study, lower rates (4.7%) of *T. annulata* infection in goat indicates that the mild to moderate susceptibility of goats to infection with *T. annulata* (43) and the possibility of goat acting as a reservoir of *T. annulata* under field conditions.

Regarding ticks infesting goats, *Rhipicephalus* spp. (namely *R. turanicus*, *R. sanguineu*, and *R. bursa* respectively were found to be the most dominating in this study, as has been also recorded in Turkey (22), Iran (44), and Iraq (45). Apart from *Rhipicephulus* spp., the second most common species of tick infesting goats werefound to be *Hyalomma* spp. Similarly, the most common tick species of goat in Iraq is *Rhipicephalus* spp. followed by the *Hyalomma* spp. (45). In opposition to our results, *Hyalomma* spp. and *Dermacentor* spp. existed only in the mountainous parts of the countries among goats (46). In consistentwith the findings of other studies (44), *D. marginatus* exhibited the lowest (4.2%) distribution in this work. This is because of a different geographical and metrological structure of the mountainous area versus semi-arid climate.

In this work, nested PCR incriminated *H. marginatum*, *R. turanicus* and *H. anatolicum* as suspected vectors for transmission of *Theileria* spp. Based on RFLP results, *H. anatolicum* was found to be infected by *T. annulata* and *T. lestoquardi*. Earlier studies, (5, 25, 42, 47) were corroborated our data. Transmission potential and infectivity of *R. turanicus* for *T. ovis* infection is in accordance with the other findings (13, 48). However, in contrast to our results, infection of *R. bursa*, *R. sanguineus* and *H. turanicum* were reported by *T. ovis* (6, 13)*.* A geographical disparity between two regions may have resulted in a better adaptation of one tick’s species to the local conditions, thus replacing with other ones. As shown in the current study, *R. turanicus* was infected by *T. lestoquardi*. The obtained results are in conformity with the finding of previous investigations, which reported that *Rhipicephalus* spp. maybe has a transmission role in small ruminant theileriosis (12). There were no molecular evidence about the role of the *R. turanicus* as a vector in the transmission of *T. lestoquardi* infection in goat. In Iran, in a similar study (47), *T. lestoquardi* DNA was isolated from *R. turanicus*, obtained from unknown infested host*.* Moreover, *R. turanicus* could be the main vector of *B. ovis* (16, 49). However, further detailed experimental studies are needed to demonstrate precisely that whether *R. turanicus* can transmit *T. lestoquardi* to goats.

In harmony with the findings in the present study, prevalence of theileriosis was higher in herds with tick infestation indicates the presence of a positive correlation between the prevalence of the disease and the presence of ticks (24, 25). In addition, from the results of this work, the difference between the prevalence of *Theileria* spp. infection in female ticks was statistically significant (P< 0.05). In agree-mentto the findings in this paper, the prevalence of *Theileria* infection has been reported to be higher in female ticks. This may be due to female ticks had many more type III acini than male ticks and *Theileria* parasites were only detected in type III acini (8, 50). These statements show why female ticks have greater *Theileria* infection prevalence than males.

## Conclusion

*Theileria ovis* is the dominant causative agent in this region but the evidence of *T. lestoquardi* and *T. annulata* infection of goats in few cases were noteworthy, as well. *Theileria annulata* was successfully transmitted from cattle to goats and vice versa by *H. a. anatolicum*.

Taking into account the possibility of occurrence of *T. annulata* infection in goats andsuccessful feeding of *H. anatolicum* on goat even a small portion, goats may be risk factors for theileriosis in cattle in areas where cattle and goats raised together as well as common competent vector coexist in the same area. Finally, *H. marginatum*, *R. turanicus* and *H. anatolicum* may play an important role in transmission of different species of *Theileria* in this area. This work attempted to determine the prevalence, vectors and molecular identification of caprine theileriosis in goats in limited region of Iran, however further studies with a large number of samples from large-scale of country are recommended.
